# Complete Genome Sequence of Shelby, a Siphophage Infecting Carbapenemase-Producing Klebsiella pneumoniae

**DOI:** 10.1128/MRA.01037-19

**Published:** 2019-09-19

**Authors:** Robert Saldana, Heather Newkirk, Mei Liu, Jason J. Gill, Jolene Ramsey

**Affiliations:** a Center for Phage Technology, Texas A&M University, College Station, Texas, USA; Queens College

## Abstract

Carbapenem-resistant Klebsiella pneumoniae, a bacterium of the family Enterobacteriaceae, is a high-priority antibiotic-resistant pathogen that causes nosocomial infections. Here, we describe the isolation and annotation of the K. pneumoniae siphophage Shelby, a T1-like siphophage encoding 78 proteins, of which 34 have a predicted function.

## ANNOUNCEMENT

Klebsiella pneumoniae, a Gram-negative bacterium, accounts for a significant proportion of hospital-acquired urinary tract infections, pneumonia, septicemias, and soft tissue infections worldwide (1). In recent years, the threat posed by K. pneumoniae has increased markedly with the emergence of strains resistant to carbapenems (2). A possible alternative to antibiotics is the use of bacteriophages. Here, we describe the genome of siphophage Shelby, which is active against K. pneumoniae strain 1776 (3).

Bacteriophage Shelby was isolated from filtered (pore size, 0.2 μm) samples of a pond in Research Park at Texas A&M University (College Station, TX). The isolation host, K. pneumoniae carbapenemase (KPC)-producing strain 1776 cured of the *bla*_KPC_ plasmid, was grown aerobically in tryptic soy broth or agar (Difco) at 37°C, and phage propagation was done using the soft agar overlay method (4). Phage genomic DNA was purified using the shotgun library preparation protocol modification of the Wizard DNA clean-up system (Promega) (5). After preparation with an Illumina TruSeq Nano low-throughput kit, phage genomic DNA was sequenced by an Illumina MiSeq v2 500-cycle 250-bp paired-end run. The 222,088 sequence reads in the index were quality controlled with FastQC (http://www.bioinformatics.babraham.ac.uk/projects/fastqc/) and trimmed with FASTX-Toolkit v0.0.14 (http://hannonlab.cshl.edu/fastx_toolkit/). Using SPAdes v3.5.0, a single contig of 18.3-fold coverage was assembled (6). Using Sanger sequencing of PCR products generated across the genome ends (forward primer, 5′-CCCATGGAGCCAGATAGTTTAAT-3′; reverse primer, 5′-ATTGATTCGTTCTCAGGATCTGG-3′), the contig was confirmed to be complete. Genes were predicted using Glimmer v3.0 (7) and MetaGeneAnnotator v1.0 (8). The absence of tRNA genes was verified by ARAGORN v2.36 (9). Functional annotation was completed with InterProScan v5.22-61 (10) and BLAST v2.2.31 with a 0.001 minimum expectation value cutoff (11). The databases used for BLAST analysis were the NCBI nonredundant database and the UniProtKB Swiss-Prot and TrEMBL databases (12). Additional tools used with default settings included TMHMM v2.0 and HHpred (with HHblits ummiclust30_2018_08 for multiple sequence alignment [MSA] generation and PDB_mmCIF70 for modeling using HH-suite v3.0) predictions (13, 14). TransTermHP v2.09 was used to annotate Rho-independent terminators (15). The annotation tools are hosted on the Center for Phage Technology Galaxy (16) and Web Apollo (17) instances (https://cpt.tamu.edu/galaxy-pub/). Phage morphology was determined by negative staining with 2% (wt/vol) uranyl acetate and viewing by transmission electron microscopy at the Texas A&M Microscopy and Imaging Center (18).

Shelby has a 49,045-bp genome with a G+C content of 50.4%. This G+C content is lower than that of its host, which is 57.5% (3). The coding density of the Shelby genome is 90.6%, and it has 78 coding sequences, with 34 having a predicted gene function. Based on analysis with progressiveMauve v2.4.0, Shelby has 85.5% and 84.9% shared nucleotide sequence identities with *Klebsiella* phage 1513 (GenBank accession number KP658157) and *Klebsiella* phage Sushi (accession number KT001920), respectively, and there are 69 similar proteins between the three (19). Shelby also has 33 proteins with BLASTp similarity to those of phage T1 (NCBI accession number NC_005833), and a similar overall genome size. Unsurprisingly, Shelby is predicted by PhageTerm to use a headful mechanism for DNA packaging (20).

### Data availability.

The genome sequence and associated data for phage Shelby were deposited under GenBank accession number MK931445, BioProject accession number PRJNA222858, SRA accession number SRR8869238, and BioSample accession number SAMN11360410.
